# Clinical Management of Suppurative Osteomyelitis, Bisphosphonate-Related Osteonecrosis, and Osteoradionecrosis: Report of Three Cases and Review of the Literature

**DOI:** 10.1155/2013/402096

**Published:** 2013-10-07

**Authors:** Eduardo Pereira Guimarães, Fernanda Rafaelly de Oliveira Pedreira, Bruno Correia Jham, Marina Lara de Carli, Alessandro Antônio Costa Pereira, João Adolfo Costa Hanemann

**Affiliations:** ^1^Department of Clinic and Surgery, School of Dentistry, Federal University of Alfenas, 37130-000 Alfenas, MG, Brazil; ^2^Oral Pathology, College of Dental Medicine-Illinois, Midwestern University, Downers Grove, IL 60515, USA; ^3^Oral Pathology, Department of Biomedical Sciences, Federal University of Alfenas, 37130-000 Alfenas, MG, Brazil; ^4^Stomatology, Department of Clinic and Surgery, School of Dentistry, Federal University of Alfenas, 37130-000 Alfenas, MG, Brazil

## Abstract

In the past, osteomyelitis was frequent and characterized by a prolonged course, treatment response uncertainty, and occasional disfigurement. Today, the disease is less common; it is believed that the decline in prevalence may be attributed to increased availability of antibiotics and improvement of overall health patterns. Currently, more common osteomyelitis variants are seen, namely, osteoradionecrosis (ORN) and bisphosphonate-related osteonecrosis of the jaws (BRONJ). Osteomyelitis, ORN, and BRONJ can present with similar symptoms, signs, and radiographic findings. However, each condition is a separate entity, with different treatment approaches. Thus, accurate diagnosis is essential for adequate management and improved patient prognosis. The aim of this paper is to report three cases of inflammatory lesions of the jaws—osteomyelitis, ORN, and BRONJ—and to discuss their etiology, clinical aspects, radiographic findings, histopathological features, treatment options, and preventive measures.

## 1. Introduction

 The osteomyelitis is an inflammatory condition of the bone, which generally begins as an infection of the marrow cavity, rapidly involves the Haversian canals, and eventually extends to the periosteum [1]. In the past, osteomyelitis was frequent and characterized by a prolonged course, treatment response uncertainty, and occasional disfigurement (due to loss of bone and teeth and resulting facial scars). Today, the disease is less common, and it is believed that the decline in prevalence may be attributed to the increased availability of antibiotics and improvement of overall health patterns [2]. Nonetheless, osteomyelitis remains a challenging disease for both clinicians and patients.

Currently, more common osteomyelitis variants are seen. ORN is one of the most serious complications in the treatment of head and neck malignancies and is defined as the ischemic necrosis of the irradiated bone, which becomes hypovascular, hypocellular, and hypoxic [3, 4]. BRONJ is one of the more recently reported serious adverse effects of bisphosphonates treatment, which are used to manage oncologic patients and to prevent fractures in osteoporosis [5, 6].

Osteomyelitis, ORN, and BRONJ can present with similar symptoms, signs, and radiographic findings. However, each condition is a separate entity, with different management approaches [7]. Thus, the aim of this paper is to report three cases of inflammatory lesions of the jaws—osteomyelitis, ORN, and BRONJ—and to discuss their etiology, clinical aspects, radiographic findings, histopathological features, treatment options, and preventive measures. Written informed consent was obtained from all the patients.

## 2. Case Reports

**Case 1**

**Figure 1:**
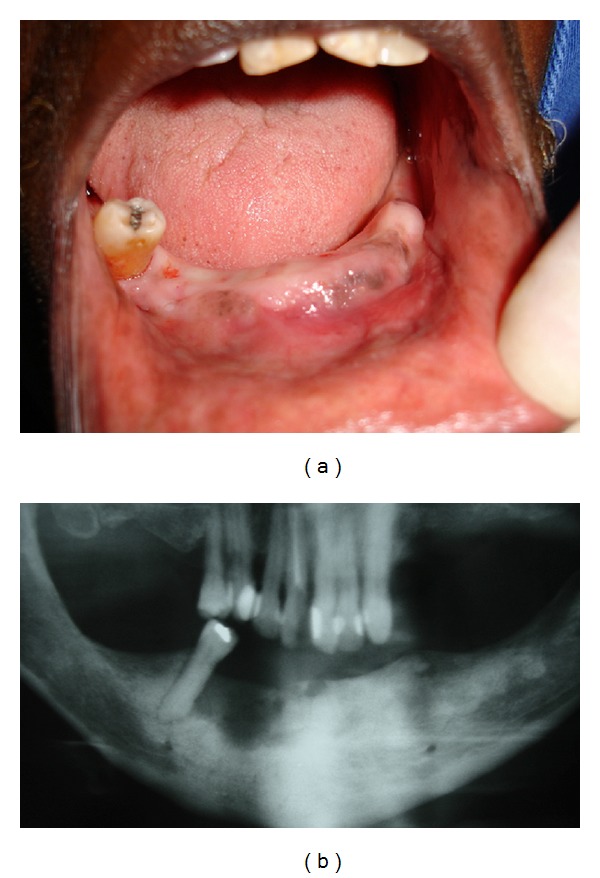
((a) and (b)) Initial clinical and radiographic features of osteomyelitis, located in the anterior mandible, consistent with necrotic bone.

**Figure 2:**
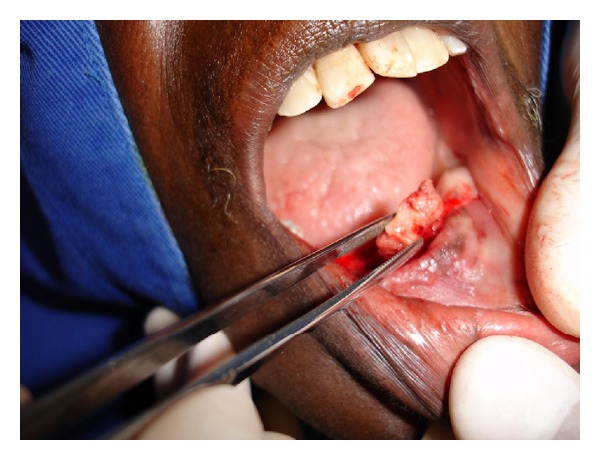
Surgical removal of bone sequestration.

**Figure 3:**
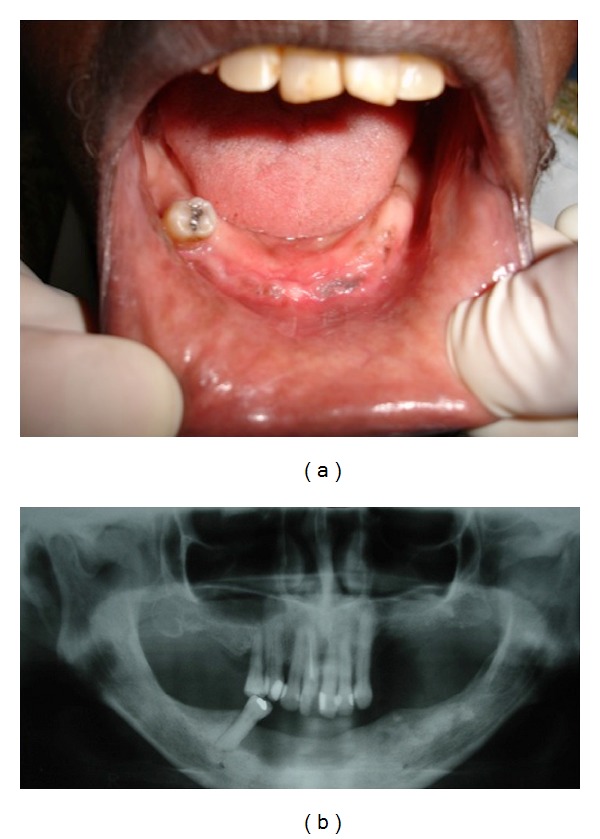
((a) and (b)) Clinical and radiographic aspects 30 days after surgery showing almost complete healing of the operated area.

A 77-year-old white female was seen at the Oral Medicine Clinic of the Federal University of Alfenas (UNIFAL-MG) with an asymptomatic, smooth surfaced, normal colored tumor on the anterior mandibular alveolar ridge, with two months evolution. A drainage point with purulent material was also present (Figure 1(a)). The patient's medical history was unremarkable and no changes were noted on extraoral examination. Radiographic examination revealed osteolysis and bone sequestration on the mandibular alveolar ridge (Figure 1(b)). Based on clinical and radiographic findings, a provisional diagnosis of osteomyelitis was rendered. The patient was given amoxicillin (500 mg, three times/day) for 15 days and subsequently underwent excision of the bone sequestrum and curettage of the granulation tissue (Figure 2). The material was submitted to histopathological examination which revealed nonviable bone and a mixed inflammatory infiltrate of lymphocytes and plasma cells, confirming the diagnosis of chronic suppurative osteomyelitis. The area healed appropriately within one month (Figures 3(a) and 3(b)). The patient has been under follow-up for 5 years with no signs of recurrence.

**Case 2**

**Figure 4:**
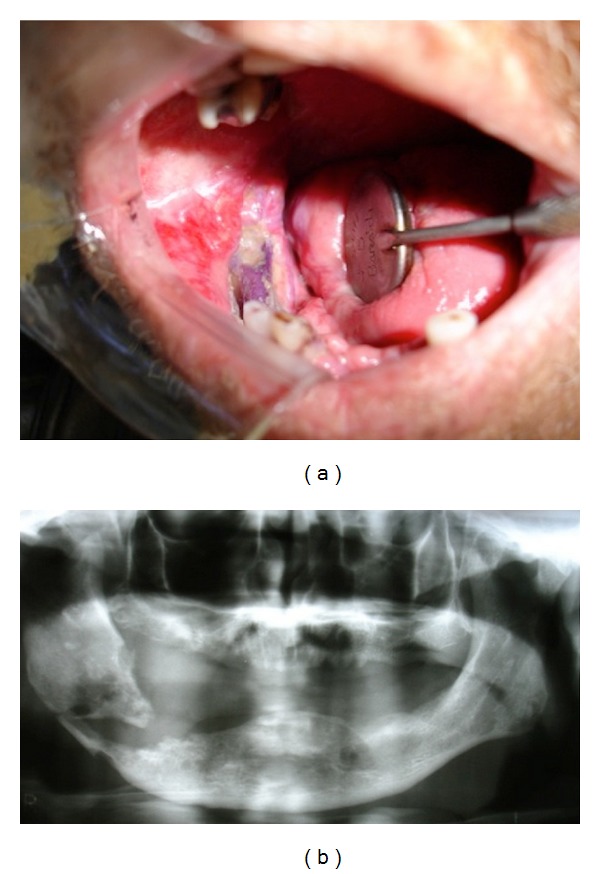
(a) Clinical aspects of osteoradionecrosis showing bone exposure of the operated area. (b) Panoramic radiograph showing pathological fracture in the mandible.

A 46-year-old male was seen at the Oral Medicine Clinic of the UNIFAL-MG with chief complaint of pain in the area of tooth no. 20. Medical history revealed a history of oral squamous cell carcinoma 15 months before. The cancer had been treated with 40 sessions of radiotherapy and 7 cycles of chemotherapy six months before. No surgical treatment and preradiotherapy dental assessment had been performed. A history of tobacco and alcohol use was also reported. Clinical examination showed splinting of anterior teeth due to periodontal disease, absence of teeth, and generalized radiation caries. Radiographic examination showed generalized bone loss. In view of the patient's unsatisfactory oral condition and due to the short period since the end of radiotherapy, a conservative approach was instituted. Preventive and restorative procedures were executed for oral health establishment. Subsequently, no. 28 and the anterior incisors, presenting extensive carious lesions with periapical involvement, were extracted under antibiotic therapy (500 mg amoxicillin, three times a day, for 10 days). After 15 days, the extraction site in the region of no. 28 failed to heal appropriately. Thus, irrigation with sodium iodide and chlorhexidine, as well as surgical debridement, was instituted. After 7 days, no improvement was noted; clinical examination revealed an ulcer with an erythematous halo and a serofibrinous pseudomembrane (Figure 4(a)). The underlying cortical bone was exposed and necrotic, and intense pain was reported. A diagnosis of ORN was established and the patient was submitted to segmental osteotomy of the involved region. At this moment, the patient showed no signs of lesion recurrence or any residual tumor. Unfortunately, primary closure was not obtained and the patient eventually developed a pathological fracture within one year (Figure 4(b)). A few months later, the patient succumbed to hepatic metastases.

**Case 3**

**Figure 5:**
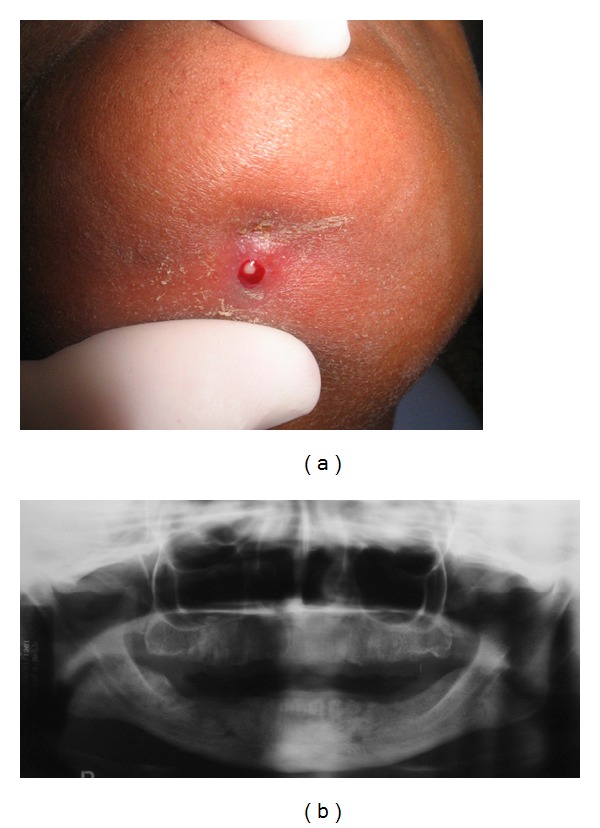
((a) and (b)) Clinical aspect of BRONJ showing purulent drainage in the submental region and absence of radiographic changes.

A 51-year-old female was referred for evaluation of a submental fistula with purulent drainage, with evolution of 7 days (Figure 5(a)). Medical history revealed breast cancer and use of pamidronate (90 mg) for the past two years. Clinical exam showed absence of all teeth and normal mucosa. No radiographic changes were observed (Figure 5(b)). Based on clinical history and findings, the diagnostic hypothesis was BRONJ. The purulent material was collected and cultured, and revealed the presence of *Staphylococcus epidermidis* with sensitivity to clindamycin. Based on microbiological analysis, the patient was administered 300 mg oral clindamycin for 35 days, and the use of pamidronate was discontinued. Following this period, fistula closure was observed, along with absence of drainage. The patient is currently being followed up with no signs of recurrence.

## 3. Discussion

 Osteomyelitis, ORN, and BRONJ are all, in general, more common in the mandible (angle and body) than in the maxilla. This likely occurs due to the mandible's increased density and less vascularized cortical plates. Also, in contrast to the maxilla, the mandible has only a single blood supply source, from the inferior alveolar neurovascular bundle [8, 9]. In agreement with the literature [10, 11], all of our cases developed in the mandible.

Local and systemic host factors are key in understanding the pathogenesis of osteomyelitis [12]. The cause of osteomyelitis is predominantly odontogenic (dental-infection related) or traumatic (fracture related) in nature. In most cases, the primary complaints are swelling, pain, or draining fistula. Other initial signs include pathologic fracture, malocclusion, sequestra, and exposed bone, fever, and trismus [9]. A wide range of organisms contributes to chronic osteomyelitis [12]. No specific microorganism is found to be a predominant etiologic agent for osteomyelitis [9]. The distinction between acute and chronic osteomyelitis is based on evolution; an acute process develops up to one month after appearance of the symptoms, while a chronic process takes longer than one month [13, 14].  Case 1 evolved for 2 months, characterizing chronic osteomyelitis. The most common findings of osteomyelitis are diffuse sclerosis, a peripheral sclerotic rim, cortical layering (involucrum), central loss of trabecular pattern with internal round radiolucent resorptive tracts, minimal jaw expansion, and reduction of the alveolar cortex on radiographic examination [8, 11], features which were also presents in our case.

The pathogenesis of ORN has not yet been fully elucidated. It is thought that radiation-generated free radicals and corresponding damage to endothelial cells lead to hypovascularity, tissue hypoxia, destruction of bone-forming cells, and marrow fibrosis [15]. Radiation may also suppress bone turnover via osteoclasis [16]. In addition, anaerobic bacteria may play a fundamental role in the pathogenesis of ORN [17]. The incidence of ORN in patients varies from 0.4% to 56% [15], but it has declined in recent decades [17]. Such decrease in presumably is due to the advent of megavoltage RT and to the increased awareness of the importance of oral health care [4]. 

ORN is typically associated with surgical extractions, which should ideally be performed 21 days before initiation of RT [15]. If absolutely necessary, post-RT extractions should be performed with minimal trauma and under antibiotic therapy. In Case 2, the patient was only seen after RT. Because no pretherapy oral treatment was instituted, post-RT surgical intervention was required. This was probably a key factor in the development of the ORN [17, 18].

Bisphosphonates are primarily effectively employed in neoplasia-related conditions, such as malignant hypercalcemia, bony metastasis, and lytic lesions of multiple myelomas [19]. Among bisphosphonates, zoledronic acid and pamidronate are most commonly employed. In addition, alendronate, risedronate, and ibandronate are frequently used for osteoporosis [20]. ONJ may occur due to enzymatic inhibition, which leads to cell toxicity induced by the medication. Patients that use intravenous bisphosphonates are more susceptible to BRONJ than those that take the medication orally. Systemic factors, such as diabetes, immunosuppression, and concomitant use of other drugs may also be involved with the development of BRONJ [21]. Most cases of BRONJ occur following extractions. Occasionally, BRONJ may spontaneously develop [22], such as in the current case. No oral infectious foci, exposed areas, or radiographic findings were present. 

Surgical therapy in association with antibiotics and anti-inflammatory drugs has been shown to be beneficial for patients with all three types of disease, with significant improvement in quality of life. Still, exacerbations may occur and regular follow-up is necessary [23–25]. In our study, two patients underwent surgical intervention for their disease. Although patient 1 evolved to cure, patient 2 developed a pathological fracture in the ORN region, highlighting that the clinical picture may exacerbate even with surgical intervention [26]. Patient 3 was managed with medications only, without the necessity for surgical intervention. Hyperbaric oxygen and platelet-rich plasma have been recommended for the treatment of both ORN and BRONJ. However, the benefits of these therapies are inconsistent in the literature [25, 27–30].

## 4. Conclusions

 Bone inflammatory diseases are important due to high morbidity and mortality rates. Even with a decline in traditional osteomyelitis, ORN and BRONJ have emerged as important secondary consequences of commonly used therapeutics. Osteomyelitis, ORN, and BRONJ may present with similar sign and symptoms; thus, it is crucial for oral health professionals to differentiate between these processes. A correct diagnosis will allow adequate management and improve patients' prognosis. Dentists also play an important role in preventing damage, by providing pretherapy care aiming to eliminate compromising or infectious foci.
